# Sleep Duration Trajectories and Body Composition in Adolescents: Prospective Birth Cohort Study

**DOI:** 10.1371/journal.pone.0152348

**Published:** 2016-03-24

**Authors:** Antônio Augusto Schäfer, Marlos Rodrigues Domingues, Darren Lawrence Dahly, Fernanda Oliveira Meller, Helen Gonçalves, Fernando César Wehrmeister, Maria Cecília Formoso Assunção

**Affiliations:** 1 Post-Graduate Program in Epidemiology, Federal University of Pelotas, Pelotas, Brazil; 2 Post-Graduate Program in Physical Education, Federal University of Pelotas, Pelotas, Brazil; 3 Department of Epidemiology and Public Health, University College Cork, Cork, Ireland; University of São Paulo, BRAZIL

## Abstract

We aimed to estimate the association between sleep duration trajectories and body composition in adolescents. We used data from participants of the 1993 Pelotas (Brazil) Birth Cohort Study who were later followed up at age 18 years (response rate of 81.3%). At the time, 3974 adolescents had complete data on body composition, which was assessed by air displacement plethysmography. Sleep duration was self-reported by participants at ages 11 and 18 years. Analyses were sex-stratified. The mean sleep duration at 11 years was 9.7 (SD 1.4) and 8.4 (SD 1.9) at 18 years. Sleep duration was dichotomized as inadequate (<8 hours/day) or adequate (≥8 hours/day). Mean body mass, fat mass, and fat-free mass indices at 18 years were 23.4 kg/m^2^ (SD 4.5), 6.1 kg/m^2^ (SD 3.9) and 17.3 kg/m^2^ (SD 2.5), respectively. Girls who reported inadequate sleep duration at 11 years of age, but adequate sleep duration at 18, on average experienced an increase in body mass index (β = 0.39 z-scores; 95% CI 0.13, 0.65), fat mass index (β = 0.30 z-scores; 95% CI 0.07, 0.53), and fat-free mass index (β = 0.24 z-scores; 95% CI 0.08, 0.39) compared to those who had adequate sleep duration at both time points. The results suggest that changes in sleep duration across adolescence may impact body composition in later adolescence and that this may differ by sex.

## Introduction

Adolescent obesity is an important public health issue worldwide [1]. In Brazil, overweight prevalence among adolescents has steadily increased over the past 30 years. In 2008–2009, 20.5% of adolescents were classified as overweight, six times the prevalence among boys and three times among girls in 1974–1975 [2]. The prevalence of obesity in the same period has increased from 0.4% to 5.9% among boys and 0.7% to 4.0% among girls [2]. The physical and psychosocial consequences of adolescent obesity have been well documented [3, 4].

The causes for the rise of the obesity epidemic are not obvious because it has biological, economic, social and cultural determinants. While the most proximal cause of excessive accumulation of body fat is a positive energy balance, this imbalance is triggered by a number of other factors. One hypothesized factor is sleep duration, which has been linked to obesity risk and body composition in several recent epidemiological studies [5–8]. Further, the increasing prevalence of overweight and/or obesity has coincided with a reduction in sleep duration in modern societies [9]. For example, data from the USA show that adults have on average reduced their sleep duration by one to two hours, and that more than a third of young adults are sleeping less than seven hours [10], a phenomenon also reported among children and adolescents [11, 12]. A study conducted with young people aged 10–19 years in the state of São Paulo, Brazil, showed that 39% of them slept eight hours or less [13].

The precise biological mechanisms that mediate the relationship between sleep and body composition are unknown. However, both laboratory and population-based studies have suggested pathways such as decreased leptin levels, elevated ghrelin levels, tiredness and increased opportunity for food intake [14, 15]. These mechanisms could lead to appetite stimulation, increased energetic intake and decreased physical activity, promoting weight gain [14, 15].

We conducted sex-stratified analyses based on evidence for sex differences in the association between sleep duration and obesity in adolescents [16, 17]. We postulated that short sleep duration during adolescence would predict BMI and fat mass in late adolescence, and that the effect would be stronger in girls compared with boys. We also hypothesized that adequate sleep duration during adolescence would be associated with fat-free mass gain, and that the effect would be stronger in boys compared with girls.

Since adolescence has been identified as a risk period for sleep-related problems [18, 19], and it is a phase characterized by changes in body composition [20], we aimed estimate the relationship between sleep duration trajectories and body mass, fat mass, and fat-free mass indices during adolescence, using data from the 1993 Birth Cohort of Pelotas, Brazil.

## Methods

### Subjects

All 5365 children born in hospitals of the urban area of Pelotas, Southern Brazil, in 1993 were recruited for a birth cohort study (n = 5265). The resultant birth cohort consisted of 5249 live births (16 declined to participate) [21]. These participants have been followed on several occasions [22]. In the present analyses we use data from two follow-up visits.

All cohort members were followed up in 2004–2005, at the age of 11 years. Those who completed the interviews, added to those known to have died, made up 87.5% (n = 4452) of the original cohort. Adolescents and their mothers were interviewed during home visits.

At the 18-year follow-up, we interviewed 4106 adolescents, for a response rate of 81.3%. More detailed information about the study can be found elsewhere [22]. In the present study we included 3974 adolescents for whom body composition measurements were available.

This study was approved by the Ethics Committee of the Medicine School of the Federal University of Pelotas in an official letter numbered 05/11. Written informed consent was obtained prior to each follow-up.

### Measurements

Weight, height, and body composition (fat mass and fat-free mass) were evaluated when participants were 18 years of age. Fat mass and fat-free mass were obtained by air displacement plethysmography (BOD POD®). We calculated their respective indices by dividing fat mass and fat-free mass (in kg) by height (in m^2^). Since fat mass and fat-free mass indices take into account the height they improve the reading in individuals with different heights [23]. Body mass index (BMI) was similarly calculated by dividing body weight (kg) by height (m) squared.

Regarding the exam, subjects wore top and shorts (spandex), and a silicone swimming cap. Weight was measured by a high precision scale (0.01 kg) part of the BOD POD® machine. Height was measured twice by trained researcher using a Harpenden metal stadiometer, to the nearest mm. According to the World Health Organization (WHO) BMI-for-age and sex reference in z score [24] normal, overweight, and obesity were defined as BMI-for-age ≤ +1 SD, > + 1 SD and > + 2 SD, respectively.

Sleep duration at 11 and 18 years old was collected by asking the adolescents two questions: "What time do you usually fall asleep on weekdays?" and "What time do you usually wake up on weekdays?" Sleep duration was calculated as the time difference between the two answers, and was categorized as <8 or ≥8 h per day, according to National Sleep Foundation’s recommendations [25].

### Statistical analyses

BMI, fat mass index and fat-free mass index were standardized as sample-specific z-scores and analyzed as continuous variables. Sleep duration trajectories were created based on the combination of sleep duration levels at 11 and 18 years of age and was classified as *always adequate* (≥8 h); *adequate-inadequate* (≥8 h, <8 h); *inadequate-adequate* (<8 h, ≥8 h); and *always inadequate* (<8 h).

Crude and adjusted analyses were performed using linear regression. Possible confounders added to the model were: maternal skin color, gestational weight gain [26], pregnancy smoking, pregnancy alcohol consumption, birth order, type of delivery, family income at birth, maternal education at birth, maternal age at birth, birth weight, physical activity at 11 years, and screen time at 11 years. Physical activity was evaluated using a questionnaire to measure commuting to and from school, and leisure time activities [27]. The questionnaire also included information on screen time. The mean time spent in front of TV, videogame, and computer (in a typical week) was noted separately for weekdays and weekends. Screen time variable was constructed by adding the weighted mean screen time (TV + videogame + computer), assigning weight 5 to weekdays and weight 2 to weekends and dividing the result by 7 to obtain the mean time in minutes per day.

All analyses were stratified by sex and performed using Stata version 12.1 (Stata Corp., College Station, Texas, USA).

## Results

Table 1 gives the participant characteristics. Two-thirds of the adolescents´ mothers had inadequate gestational weight gain (66.0%), and almost one-third reported smoking during pregnancy (32.8%) and had caesarean sections (31.1%). The median physical activity at 11 years was 285 minutes per week (IQR: 140 to 540). Also, the mean of maternal education at birth was almost seven years (SD 3.5) and adolescent screen time at 11 years was 4.3 hours per day (SD 2.7). The average sleep duration at 11 and 18 years was 9.7 (SD 1.4) and 8.4 (SD 1.9) hours, respectively. In relation to the outcomes studied, the means of BMI, fat mass index and fat-free mass index were 23.4 kg/m^2^ (SD 4.5), 6.1 kg/m^2^ (SD 3.9) and 17.3 kg/m^2^ (SD 2.5), respectively.

**Table 1:** Description of variables studied by sex. 1993 Pelotas (Brazil) Birth Cohort Study (n = 3974).

**Categorical variables**	**Total**			**Male**			**Female**		
	**n (%)**			**n (%)**			**n (%)**		
**Maternal skin color**									
White	3057 (77.0)			1527 (77.4)			1530 (76.5)		
Black	735 (18.5)			352 (17.8)			383 (19.2)		
Other	180 (4.5)			94 (4.8)			86 (4.3)		
**Gestational weight gain**									
Adequate	1291 (34.0)			642 (34.2)			649 (33.8)		
Inadequate	2509 (66.0)			1237 (65.8)			1272 (66.2)		
**Pregnancy smoking**									
No	2671 (67.2)			1341 (68.0)			1330 (66.5)		
Yes	1303 (32.8)			632 (32.0)			671 (33.5)		
**Pregnancy alcohol consumption**									
No	3773 (94.9)			1886 (95.6)			1887 (94.3)		
Yes	201 (5.1)			87 (4.4)			114 (5.7)		
**Birth order**									
Firstborn	1580 (39.8)			773 (39.2)			807 (40.3)		
Second	1190 (30.0)			591 (30.0)			599 (30.0)		
Third	628 (15.8)			300 (15.2)			328 (16.4)		
Forth or later	573 (14.4)			307 (15.6)			266 (13.3)		
**Type of delivery**									
Normal	2738 (68.9)			1360 (68.9)			1378 (68.9)		
Caesarean	1236 (31.1)			613 (31.1)			623 (31.1)		
**BMI at 11 years**									
Normal	2952 (76.6)			1412 (74.5)			1540 (78.7)		
Overweight	448 (11.6)			189 (10.0)			259 (13.2)				
Obesity	453 (11.8)			294 (15.5)			159 (8.1)		
**BMI at 18 years**									
Normal	2881 (72.7)			1467 (74.6)			1414 (70.9)		
Overweight	680 (17.2)			318 (16.2)			362 (18.1)		
Obesity	400 (10.1)			180 (9.2)			220 (11.0)		
**Continuous variables**	**Total**			**Male**			**Female**		
	**n**	**Median**	**IQR**	**n**	**Median**	**IQR**	**n**	**Median**	**IQR**
**Family income (MMW)**	3907	2.6	1.5–4.6	1945	2.5	1.5–4.5	1962	2.9	1.5–4.8
**Physical activity at 11 years (minutes/week)**	3733	285	140–540	1846	370	180–660	1887	220	110–420
	**n**	**Mean**	**SD**	**n**	**Mean**	**SD**	**n**	**Mean**	**SD**
**Maternal education (completed years)**	3967	6.8	3.5	1969	6.8	3.5	1998	6.8	3.5
**Maternal age at birth (years)**	3973	26.1	6.4	1972	26.1	6.5	2001	26.1	6.3
**Birth weight (grams)**	3969	3181.8	527.7	1970	3249.2	534.7	1999	3115.4	512.2
**Screen time at 11 years (hours/day)**	3844	4.3	2.7	1885	4.5	2.9	1959	4.2	2.5
**Sleep duration at 11 years (hours)**	3849	9.7	1.4	1889	9.6	1.4	1960	9.7	1.4
**Sleep duration at 18 years (hours)**	3955	8.4	1.9	1962	8.1	1.8	1993	8.8	1.9
**BMI (kg/m**^**2**^**)**	3974	23.4	4.5	1973	23.4	4.2	2001	23.5	4.8
**Fat mass index (kg/m**^**2**^**)**	3974	6.1	3.9	1973	4.2	3.1	2001	8.0	3.6
**Fat-free mass index (kg/m**^**2**^**)**	3974	17.3	2.5	1973	19.1	1.9	2001	15.5	1.6

Maximum percentage of unknown observations: (n = 241; 6.1%) for the physical activity at 11 years variable.

MMW: Monthly minimum wages.

SD: Standard deviation.

IQR: Interquartile range.

BMI: Body mass index.

**Fig 1:**
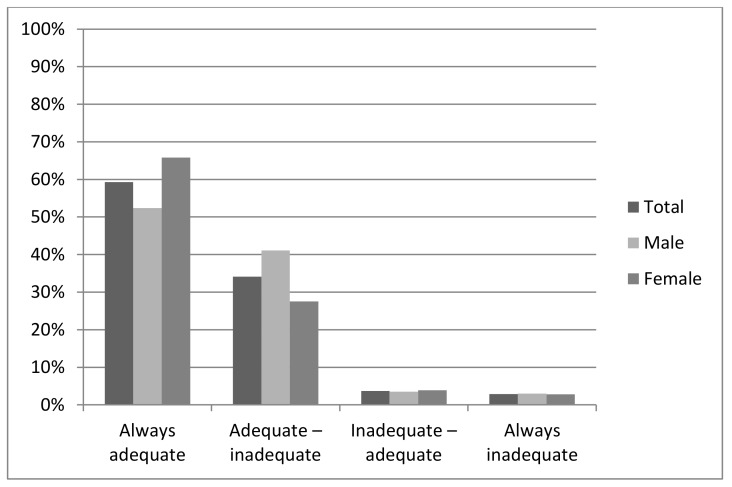
Prevalence of sleep duration trajectories. 1993 Pelotas (Brazil) Birth Cohort Study.

We observed that more than one-third of the adolescents reported adequate sleep at 11 years of age but inadequate sleep at 18 (41.1% and 27.5% among males and females, respectively). On the other hand, only 3.7% of the adolescents improved their sleep duration (Fig 1).

Crude and adjusted analyses of the association between sleep duration trajectories during adolescence and the outcomes stratified by sex are shown in the Table 2. In the adjusted models, change in sleep duration remained associated with BMI and fat mass index in females. Girls who increased their sleep duration from 11 to 18 years of age showed an increase of 0.39 z-scores (95% CI 0.13, 0.65) and 0.30 z-scores (95% CI 0.07, 0.53) in BMI and fat mass index, respectively, when compared to those who always had adequate sleep duration. Fat-free mass index was associated with change in sleep duration in both sexes. Boys who went from adequate to inadequate sleep duration during adolescence had an increase of 0.09 z-scores (95% CI 0.01, 0.16) in fat-free mass index, whereas girls who improved their sleep duration from 11 to 18 years of age showed an increase of 0.24 z-scores (95% CI 0.08, 0.39) in fat-free mass index compared to adolescents who had adequate sleep duration in both follow-ups.

**Table 2:** Crude and adjusted analyses of association between sleep duration trajectories (between 11 and 18 years) and outcomes (in z-scores) stratified by sex. 1993 Pelotas (Brazil) Birth Cohort Study.

	Crude analyses						Adjusted analyses*					
	Male			Female			Male			Female		
	n	β (95% CI)	p^†^	n	β (95% CI)	p^†^	β (95% CI)	p^†^	R^2^^Ɨ^	β (95% CI)	p^†^	R^2^^Ɨ^
**BMI**												
Sleep duration trajectories			0.041			0.015		0.203	0.03		0.029	0.03
Always adequate	982	Reference		1279	Reference		Reference			Reference		
Adequate–inadequate	775	0.09 (0.001;0.18)		543	-0.03 (-0.14;0.08)		0.08 (-0.01;0.17)			-0.01 (-0.12;0.10)		
Inadequate–adequate	65	0.18 (-0.06;0.42)		76	0.39 (0.14;0.63)		0.13 (-0.13;0.39)			0.39 (0.13;0.65)		
Always inadequate	56	0.27 (0.01;0.52)		54	-0.02 (-0.31;0.26)		0.19 (-0.07;0.45)			-0.03 (-0.33;0.26)		
**Fat mass index**												
Sleep duration trajectories			0.037			0.034		0.174	0.03		0.049	0.02
Always adequate	982	Reference		1279	Reference		Reference			Reference		
Adequate–inadequate	775	0.04 (-0.03;0.12)		543	-0.03 (-0.12;0.06)		0.03 (-0.05;0.11)			-0.03 (-0.13;0.07)		
Inadequate–adequate	65	0.25 (0.04;0.45)		76	0.30 (0.09;0.52)		0.23 (0.0003;0.45)			0.30 (0.07;0.53)		
Always inadequate	56	0.20 (-0.58;-0.47)		54	-0.02 (0.44;0.54)		0.13 (-0.09;0.36)			-0.05 (-0.31; 0.21)		
**Fat-free mass index**												
Sleep duration trajectories			0.020			0.026		0.023	0.03		0.030	0.06
Always–adequate	982	Reference		1279	Reference		Reference			Reference		
Adequate–inadequate	775	0.10 (0.03;0.17)		543	-0.01 (-0.07;0.06)		0.09 (0.01;0.16)			0.03 (-0.04;0.10)		
Inadequate–adequate	65	-0.05 (-0.24;0.13)		76	0.23 (0.08;0.38)		-0.11 (-0.32;0.09)			0.24 (0.08;0.39)		
Always inadequate	56	0.17 (-0.03;0.36)		54	-0.005 (-0.18;0.17)		0.14 (-0.07;0.34)			0.03 (-0.15;0.20)		

BMI: Body mass index; CI: Confidence interval.

*For family income, maternal education, maternal skin colour, maternal age at birth, gestational weight gain, pregnancy smoking, pregnancy alcohol consumption, birth order, type of delivery, birth weight, physical activity at 11 years, and screen time at 11 years.

^†^Wald test for heterogeneity.

^Ɨ^Adjusted R-squared.

## Discussion

Among girls, we found an association between sleep duration and BMI, fat mass index and fat-free mass index. In contrast, in boys, we found a very weak association between sleep duration and fat-free mass index. Previous studies have been inconsistent. Another cohort study including adolescents found no longitudinal association between sleep duration and body fat percentage in both sexes; however, in the cross-sectional analyses at 17 years a positive association was found among girls only [28]. Similarly, two cross-sectional studies showed a positive association between sleep duration and body fat percentage in female adolescents only [29, 30]. On the other hand, a longitudinal study found no relationship between total sleep and BMI or body fat percentage in either boys and girls [31].

To the best of our knowledge, this is the first study that examined the relationship between sleep duration and fat-free mass in adolescents. Sleep is an active process in the brain that is necessary for restorative functions and hormone secretion, particularly during development period [32], and thus it may play an important role in the accrual of fat-free mass. More studies are necessary to better understand this finding.

Storfer-Isser et al. performed an exploratory analysis to understand sex differences in the association between sleep duration and BMI finding that morning leptin levels were significantly higher among girls than boys [17]. Another important point to be considered is the sex differences in the physical and sedentary behaviors. During the adolescence, for example, physical activity levels decrease most notably among girls [33] and regular exercise may help promote suffıcient sleep [34].

The sex distinctions observed in our study may be due to the differences in the physiology of puberty in males and females as they relate to changes in body composition [35]. We have hypothesized that the effect of sleep duration might be masked in girls because they suffer greater changes in fat mass during adolescence compared to boys [36]. During puberty, boys experience rapid increases in fat-free mass and reduced fat mass, whereas girls gain considerable amounts of fat but relatively little fat-free mass. These differences may be largely due to hormones secretion as testosterone and oestrogen. Sex steroid hormones play important roles in the accumulation, metabolism and distribution of adipose tissue [37]. For example, testosterone and oestrogen facilitate fat deposition in the abdominal and gluteo-femoral regions, respectively [38]. Testosterone is also important for the increase in lean mass that occurs during puberty, especially in boys [39].

The strengths of our study include the prospective design, since there are few studies with adolescents based on longitudinal analyses; the large sample size; the high response rates; and the utilization of air displacement plethysmography as method for evaluating fat mass and fat-free mass.

Limitations include potential selection biases due to loss to follow-up. As described in detail previously [22], at 18-year-old-follow-up, participants with lower socioeconomic status, a worse nutritional profile, and those whose mothers had no schooling were less likely to be followed up. It is important to highlight, however, that the magnitude of these differences is modest, thus minimizing the probability of bias [22]. Another possible limitation is the methodology used to estimate sleep duration. However, a study of high school adolescents in the USA found high correlations between actigraphy and self-reported bedtimes (r = 0.70) and wake-up times (r = 0.77) during weekdays [40]. Thus, even with limitations on the accurate quantification of sleep time, it seems that our measure is good enough to discriminate the participants according to their sleep duration. Additionally, it is possible that a measurement error might have given rise to non-differential misclassification and the associations between sleep and body composition may be underestimated. Finally, although the differences in sleep duration at 11 years might reflect differences in pubertal development, and differences in developmental tempo will be related to later body composition outcomes, we do not have information of pubertal status at 11-year follow-up.

In conclusion, our findings suggest sex differences in the association between sleep duration and body composition in adolescents. Girls who improved their sleep duration during adolescence showed higher BMI, fat mass index and fat-free mass index compared to those who always had adequate sleep duration. Longitudinal studies are also useful for better understanding this relationship in adolescence. We recommend further research using valid and accurate measurements of sleep duration and body composition.
